# An Early Reduction in Treg Cells Correlates with Enhanced Local Inflammation in Cutaneous Leishmaniasis in CCR6-Deficient Mice

**DOI:** 10.1371/journal.pone.0044499

**Published:** 2012-09-28

**Authors:** Thomas Barth, Dominic Schmidt, Catherine Botteron, Trang T. T. Nguyen, Uwe Ritter, Daniela N. Männel, Anja Lechner

**Affiliations:** 1 Institute of Immunology, University of Regensburg, Regensburg, Germany; 2 Institute of Pediatrics, University of Regensburg, Regensburg, Germany; INSERM U1094, University of Limoges School of Medicine, France

## Abstract

Resistance to *Leishmania major* infection is dependent on the development of a cell-mediated Th1 immune response in resistant C57BL/6 mice whereas Th2-prone BALB/c mice develop non-healing lesions after infection. The chemokine receptor CCR6 is shared by anti-inflammatory regulatory T cells and pro-inflammatory Th17 cells. In a recent study we showed that C57BL/6 mice deficient in CCR6 exhibited enhanced footpad swelling and impaired T helper cell migration indicated by reduced recruitment of total T helper cells into the skin after infection and a reduced delayed type hypersensitivity reaction. Based on these findings we tested whether the lack of CCR6 alters Treg or Th17 cell responses during the course of *Leishmania major* infection. When we analyzed T cell subsets in the lymph nodes of CCR6-deficient mice, Th17 cell numbers were not different. However, reduced numbers of Treg cells paralleled with a stronger IFNγ response. Furthermore, the early increase in IFNγ-producing cells correlated with increased local tissue inflammation at later time points. Our data indicate an important role of CCR6 for Treg cells and a redundant role for Th17 cells in a Th1 cell-driven anti-parasitic immune response against *Leishmania major* parasites in resistant C57BL/6 mice.

## Introduction

During immune surveillance T cells constantly migrate through the body searching for antigen and aiming to get activated by antigen presenting cells. The trafficking process is coordinated by chemokines expressed in lymphoid and non-lymphoid tissues that allow T cells to reach their target tissue and to become functional effector cells. Thus, T cells acquire different patterns of chemokine receptors during their life span dependent on their activation state [1], [2], [3]. One of those activation-induced chemokine receptors is CCR6. CCR6 is expressed on naïve and memory B cells, immature dendritic cells and T cells that exhibit a memory cell phenotype [4], [5], [6]. More recent data identified CCR6 on T helper type (Th) 17 and Foxp3^+^ regulatory T (Treg) cells; two T cell subtypes with opposite functions sharing one chemokine receptor [7], [8], [9]. CCR6 is considered to be an important receptor guiding effector T cells into inflamed tissue. A high plasticity of these T cell subtypes has been demonstrated by *in vitro* studies [10]. Environmental conditions determine whether the Treg cell phenotype remains stable or shifts towards a pro-inflammatory Th17 phenotype [11], [12]. Under steady state conditions the anti-inflammatory Treg cell phenotype is stabilized. Inflammatory conditions favor the Th17 phenotype. Thus, CCR6^+^ T cells play a central role for balancing regulatory and inflammatory processes during homeostasis and inflammation [13].

Murine leishmaniasis has expanded our understanding of Th cell differentiation over the last three decades. The different clinical outcome of the human disease can be monitored in mice by the use of different mouse strains. In resistant C57BL/6 mice the self-healing cutaneous form is characterized by the development of a protective Th1 immune response. In BALB/c mice an IL-4-driven Th2 response predominates, leading to uncontrolled infection [14]. Preventing the complete elimination of leishmania parasites, Foxp3^+^ Treg cells have been demonstrated to confer protection against secondary *Leishmania major (L. major)* infections in resistant C57BL/6 mice [15], [16]. Th17 cells have been studied in susceptible BALB/c mice [17]. It turned out that Th17 cells contribute to the worsening of the disease by creating an inflammatory milieu at the site of infection leading to the recruitment of granulocytes. However, their role in resistant mice still needs to be elucidated.

Our previous data obtained in C57BL/6 CCR6-deficient (B6.CCR6^−/−^) mice showed an increase in footpad swelling during the primary as well as the secondary *L*. *major* infection and impaired Th cell functionality [18]. The latter was reflected by reduced Th cell recruitment to the site of infection, absence of a delayed type hypersensitivity reaction, and enhanced susceptibility of mice to secondary infection. However, B6.CCR6^−/−^ mice were able to resolve the infection with the same kinetics as wild type (B6.WT) mice. Macrophages still proved to be functional in B6.CCR6^−/−^ mice and might be the ultimate effector cells that ensure control of parasites. Based on the fact that CCR6 is a receptor shared by Th17 and Treg cells, we hypothesized that CCR6 deficiency affects the establishment of functional Treg and Th17 cell immune responses leading to enhanced local inflammation during primary infection. After subcutaneous (s.c.) infection we monitored the recruitment of both Th cell subtypes in B6.CCR6^−/−^ and B6.WT mice. As protective Th1-mediated immunity against the protozoan infection is established early during infection, we characterized T cell responses in the draining lymph node during the first 14 days of infection. Additionally, we analyzed the local immune reaction in the initial and at the peak phase of infection. CCR6 deficiency did not affect Th17 cell numbers in skin-draining lymph nodes of infected mice but led to a reduction in Treg cells. The reduction in Treg cell numbers was paralleled by an increased frequency of CD8^+^ IFNγ-producing cells. Enhanced footpad swelling also indicated that B6.CCR6^−/−^ mice reacted stronger to *L. major* infection than B6.WT mice. Inflammatory T cells were more frequent in the skin of B6.CCR6^−/−^ mice early after infection. They seem to condition the infected tissue towards enhanced inflammation, thus, initiating the recruitment of potentially tissue destructive granulocytes at later time points. Our data argue for a distinct role of CCR6 for the regulation of the inflammatory response after *L. major* infection, while being dispensable for Th17 cells in resistant C57BL/6 mice.

## Materials and Methods

### Ethics Statement

All animal protocols were performed according to German guidelines on the ethical use of animals and approved by the Animal Health and Care Committee of the University of Regensburg and the Regierung der Oberpfalz (54–2532.1–04/10). All mice were housed at the animal facilities of the University of Regensburg (Germany). C57BL/6 control mice were purchased from Janvier S.A.S. (Le Genest-St-Isle, France). The B6.129P2-Ccr6tm1Dgen/J mouse strain (The Jackson Laboratory; Bar Harbor, Maine, US) was backcrossed on the C57/BL6 background for at least 7 generations. The gene deficiency was confirmed by PCR according to the standard genotyping protocol. Female mice of 8–16 weeks of age were used.

### *L. major* Propagation, Infection of Mice, and Quantification of Parasites

*L. major* promastigotes (MHOM/IL/81/FE/BNI) [19] were propagated *in vitro* in blood agar cultures as described [20]. The virulence of the isolate was maintained by monthly passage through BALB/c mice. For infection, stationary-phase promastigotes were harvested and mice were infected s.c. into one or two hind footpads with 3×10^6^ promastigotes of the third to seventh *in vitro* passage. The increase of the lesion size was monitored weekly and determined by the formula ([thickness of infected right footpad − thickness of left footpad]: thickness of left footpad) × 100.

For detection of parasites footpads were dissected and genomic DNA was isolated using the Wizard genomic DNA purification kit according to the manufacturer’s instructions (Promega, Mannheim, Germany). Genomic DNA isolated from 1×10^6^ mouse splenocytes combined with 1×10^5^ *L. major* promastigotes was used as a standard. Quantification of leishmania-specific and mouse DNA was performed as described previously [21] with the modification that the specific amplicon was detected by SYBR Green fluorescence using the Absolute QPCR SYBR Green fluorescein mix (Thermo Fisher Scientific, Schwerte, Germany). Quantitative real-time PCR was conducted on an iQ5 instrument (Biorad, Munich, Germany).

### Antibodies and Reagents

The following antibodies were used for phenotyping of lymph node and footpad cells: rat anti-mouse CD4 mAb (clone RM4-5), rat anti-mouse CD8 mAb (clone 53-6.7), rat anti-mouse IL-17A mAb (clone TC11-18H10.1), rat anti-rat/mouse Foxp3 mAb (clone FJK-16s), hamster anti-mouse CD11b mAb (clone M1/70), rat anti-mouse F4/80 mAb (clone BM8) purchased from eBioscience (Frankfurt, Germany) and hamster anti-mouse CD62L mAb (clone Mel-14), rat anti-mouse CD25 (clone PC-51), rat anti-mouse IFNγ mAb (clone XMG1.2), rat anti-mouse CD103 (clone M290), rat anti-mouse GR1 mAb (clone RB6-8C5), mouse anti-BrdU mAb (B44) obtained from BD Biosciences (Heidelberg, Germany). Rat anti-mouse CCR6 mAb (clone 140706) was purchased from R&D Systems (Wiesbaden, Germany). Isotype matched control IgG was purchased at BD Biosciences. *L. major* promastigotes were objected to four freeze–thaw cycles and used as *L. major* antigen (LAg) for T cell stimulation assays.

### Experimental Setup, Preparation Cells, and FACS Analysis

Unless otherwise indicated, on day (d) 0 mice (n = 3–4 per time point and genotype) were infected with 3×10^6^ promastigotes of the same parasite preparation. Mice were sacrificed on d 3, 6, 10, 14, or 28 post infection (p.i.). Lymph nodes, spleen, bone marrow, and footpads were collected at the indicated days and single cells were isolated. Skin-draining popliteal lymph nodes were passed through a 40-µm cell strainer (BD Biosciences) to obtain single-cell suspensions. Footpads were digested with collagenase D (1 mg/ml; Roche, Mannheim, Germany) and DNase I (10 µg/ml; Sigma-Aldrich, Munich, Germany) in HBSS (plus Ca^2+^, Mg^2+^; PAA Laboratories GmbH, Coelbe, Germany) at 37°C for 30 min. The reaction was stopped with 5 mM EDTA (Merck, Darmstadt, Germany) at 37°C for 10 min and the digested tissue was passed through a 200 µm stainless steel sieve. For isolation of bone marrow cells, femurs were flushed with ice-cold PBS. Cells were washed twice. The number of viable cells was determined by trypan blue exclusion. If possible, 2×10^6^ lymph node cells were stained with the indicated antibodies in staining buffer (PBS containing 2% FCS) for flow cytometry. Data was collected on the LSR II flow cytometer (BD Biosciences) and analyzed using the DIVA and CellQuest software (BD Biosciences). All staining profiles were based on live-gated cells, as determined by forward and side scatter properties.

### Intracellular Cytokine Staining and Treg Cell Staining

For intracellular cytokine staining, 4×10^5^ lymph node cells from individual mice were incubated in triplicates with 50 ng/ml phorbol 12-myristate 13-acetate (PMA, Sigma-Aldrich, Munich, Germany) and 1 µM ionomycin (Sigma-Aldrich) in the presence of brefeldin A (BFA, 5 µg/ml, Sigma-Aldrich) in 200 µl complete T cell media (RPMI 1640 supplemented with 10% FCS, 50 µM β-mercaptoethanol, 2 mM L-glutamine, 100 U/ml penicillin and 100 µg/ml streptomycin (PAN Biotech, Aidenbach, Germany)) for 4 h at 37°C before staining. For each staining cells of 3 cultures were pooled to obtain a sufficient number of cells for cytokine analysis. Staining was performed with the intracellular cytokine staining kit (eBioscience) according to the manufacturer’s instructions. For Treg cell staining freshly isolated cells from individual mice were used. The staining was performed using the Foxp3 staining set (eBioscience).

### *In vivo* Proliferation Assay

Mice were infected with *L. major* promastigotes for the indicated periods of time. Three days before finishing the experiment, mice received BrdU (800 µg/ml) in the drinking water. Draining lymph nodes were harvested at d 0, 3, 6, 10, and 14 after infection and single-cell suspensions were prepared. Cell surface staining was performed. Incorporated BrdU was detected with the BrdU Flow Kit (BD Biosciences) according to the manufacturer’s instructions. Proliferation of either CD4^+^ or CD8^+^ cells was assayed by the presence of BrdU fluorescence in dividing cells.

### Analysis of T Cell Proliferation *in vitro*

Lymph node cells were labeled with CFSE (5(6)-Carboxyfluorescein diacetate *N*-succinimidyl ester, Invitrogen, Karlsruhe, Germany) at a final concentration of 2 µM. Cells (5×10^5^) from individual mice were cultured in triplicates in complete T cell media and stimulated with LAg of 1.5×10^6^ promastigotes or left untreated. Three cultures were pooled for proliferation analysis assayed by flow cytometry.

### Quantification of Cytokine mRNA

For analysis of gene expression lymph nodes were harvested at the indicated time points after infection and RNA was isolated from total lymph nodes using the NucleoSpin RNAII isolation kit (Macherey-Nagel, Düren, Germany) following the manufacturer’s instructions. Total RNA (200 ng) was transcribed into cDNA using M-MLV reverse transcriptase system (Promega). Quantitative RT-PCR was performed on the iQ5 instrument (Biorad). Quantification was performed using the comparative threshold cycle method comparing cytokine specific transcripts relatively to the housekeeping gene β-actin by the equation 2^−Δct^. The following primers were used: β-actin (for 5′- AGA GGG AAA TCG TGC GTG AC-3′, rev 5′-CAA TAG TGA TGA CTT GGC CGT-3′), CCL20 (for 5′-TGC TAT CAT CTT TCA CAC GA-3′, rev 5′-ATC TTC TTG ACT CTT AGG CTG-3′), p35 (for 5′-TGT CAA TCA CGC TAC CTC CTC-3′, rev 5′-TTT TCT CTG GCC GTC TTC AC-3′), p40 (for 5′-TCA GGG ACA TCA T A AAC CA-3′, rev 5′-CTT TCT GGT TAC ACC CCT CCT-3′), p19 (for 5′-CAA GGA CTC AAG GAC AAC AGC-3′, rev 5′-ATC CTC TGG CTG GAG GAG TT-3′), and IL-4 (for 5′-ACA GGA GAA GGG ACG CCA T-3′, rev 5′-GAA GCC CTA CAG ACG AGC TCA-3′).

### Cytokine ELISA

Culture supernatants of proliferation assays were collected and IL-10 and IL-17 levels were measured by ELISA using the DuoSet ELISA development system according to the manufacturer’s instructions (R&D Systems).

### Statistical Analysis

Data of two groups were analyzed by unpaired *Student’s* t-test. Multi-parametric analysis was performed using ANOVA. Data represent the mean values ± SEM, p values <0.05 were considered significant.

## Results

### Following *L. major* Infection B6.CCR6^−/−^ Mice Showed an Early Increase in Inflammatory T Cells in the Skin and a more Pronounced Footpad Swelling Later on

**Figure 1:**
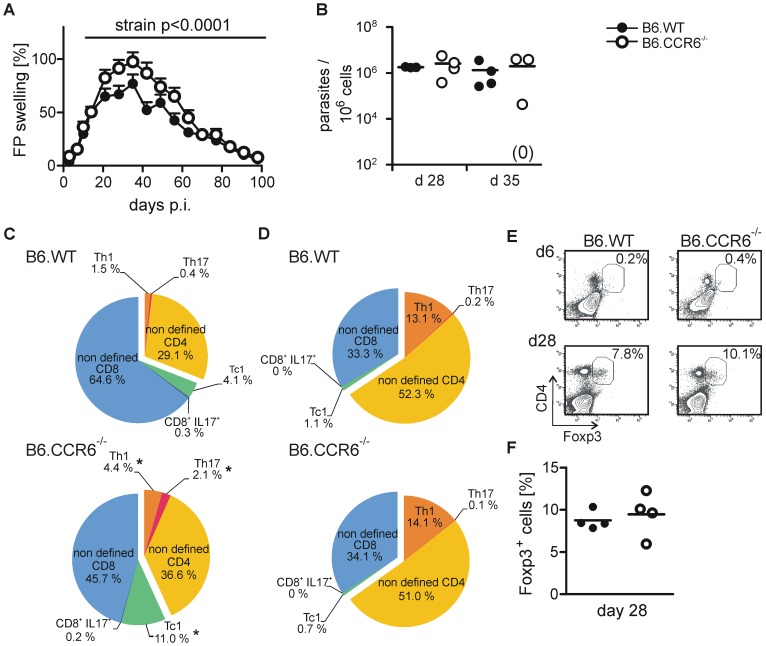
Enhanced footpad swelling correlates with an early increase in inflammatory T cells in the lesion in CCR6^−/−^ mice. Mice were infected s.c. with 3×10^6^ *L. major* promastigotes. (A) The lesion size was monitored over a period of 98 days. The relative increase in lesion size is shown (n = 20 mice, pooled data from two independent experiments). (B) Cells were isolated from footpad lesions of B6.WT or B6.CCR6^−/−^ mice 4 and 5 weeks after infection. Parasite load was determined by quantitative PCR. The number of parasites per 10^6^ mouse cells is shown (each dot represents one individual mouse, the horizontal bar indicates the mean; n = 3–4 mice per time point and genotype, data represent one experiment). (C-F) T cell composition at the site of infection is shown. (C–D) Cells were stimulated with PMA and ionomycin in the presence of BFA and stained for CD4, CD8, IFNγ, and IL-17. Live cells were gated on CD4^+^ or CD8^+^ cells and further subdivided into IFNγ^+^ and IL-17^+^ cells. The composition of cytokine producing T cells in the lesions was determined as follows: the total level of CD4^+^ and CD8^+^ cells was set to 100%. The relative frequencies of IFNγ^+^, IL-17^+^, or cells that did not produce one of the two cytokines (non-defined) within the CD4 or CD8 cell gate on d 6 (B) and d 28 (C) are shown. (E–F) Cells isolated from the footpads were stained for CD4, Foxp3, CD25, and CD103. Live cells were gated based on the forward and side scatter properties. The region indicates CD4^+^ Foxp3^+^ events. (E) Representative dot blots showing the percentage of Foxp3^+^ CD4^+^ population on d 6 (upper panel) and d 28 (lower panel) after infection are presented. (F) Cells were gated on CD4^+^ cells and the mean frequency of Foxp3^+^ cells on d 28 was determined (each dot represents an individual mouse, horizontal line represents the mean; n = 4 mice per time point and genotype of one experiment).

Four weeks after infection local tissue inflammation reached its peak in C57BL/6 mice. At that time point footpad swelling was enhanced in B6.CCR6^−/−^ compared to B6.WT mice (Figure 1A). During the observation period of 28 days, the parasite load did not differ significantly in lymph nodes of infected B6.WT and B6.CCR6^−/−^ mice (Table 1). No parasites were detected in the spleen of the two strains at any time point tested. Sporadically, few parasites were measured in the bone marrow. Also at the site of infection, the clearance of *L. major* parasites was not different between B6.WT and B6.CCR6^−/−^ mice on day 28 [1,779,659 (±53,407) parasites in B6.WT mice and 2,565,096 (±1,927,034) parasites in B6.CCR6^−/−^ mice] and on d 35 [1,331,410 (±1,312,309) parasites in B6.WT mice and 1.955.364 (±1,934,845) parasites in B6.CCR6^−/−^ mice, Figure 1B]. Since we detected a comparable parasite load in B6.CCR6^−/−^ and B6.WT mice, our data pointed to a distinct role for CCR6 in the regulation of the inflammatory response.

**Table 1:** Parasite load in lymph node and bone marrow of B6.WT and B6.CCR6^−/−^ mice.

	lymph node^a^		bone marrow^a^	
day p.i.	B6.WT	B6.CCR6^−/−^	B6.WT	B6.CCR6^−/−^
**3**	690 (±770)	210 (±300)	n.d b	n.d b
**6**	250,300 (±196,860)	216,180 (±206,180)	n.d b	n.d b
**10**	848,410 (±545,750)	43,630 (±61,700)	1^c^ (±1)	1,668^d^ (±1,890)
**28**	755,640 (±983,750)	136,000 (±128,570)	3^c^ (±4)	45^d^ (±45)

Mice were infected with *L. major* and the parasite load in different organs was quantified at the indicated time points by real-time PCR. The mean values of parasites (± SEM) per 10^6^ cells are presented.

a The experiment was performed with 3 mice per time point and genotype once.

b n.d., not detectable,

c parasites were detected in one out of three mice,

d parasites were detected in two out of three mice.

To test this assumption we monitored T cell subtypes in the footpad lesions early after infection (d 6) and at the time point of maximum swelling (d28). IFNγ-producing CD4 (Th1) as well as CD8 (Tc1) cells were more abundant in B6.CCR6^−/−^ mice than in B6.WT mice on day 6 (Figure 1C, Figure S1A). Although present in low numbers, the frequency of Th17 cells was increased in B6.CCR6^−/−^ compared to B6.WT mice in the early phase of infection. While the overall recruitment of CD4 T cells into the skin was reduced in CCR6-deficient mice [18] cytokine producing cells were more frequent indicating that the tendency towards inflammation was set early after infection in B6.CCR6^−/−^ mice. Four weeks after infection the predominant T cell population at the site of infection was the Th1 cell subtype while Th17 and Tc1 cells were rather rare (Figure 1D, Figure S1A). At that time point the frequency of IFNγ-producing CD4 cells was not different between the two strains. Treg cells were recruited to the skin later (Figure 1E). We detected a significant, but equal, frequency of CD4^+^Foxp3^+^ cells on day 28 after infection in both strains [8.75% (±0.55) for B6.WT mice and 9.50% (±1.31) for CCR6^−/−^ mice, Figure 1F]. Lesion-derived Treg cells of B6.WT or B6.CCR6^−/−^ mice showed no difference in expression of CD25 and the peripheral tissue homing marker CD103 (Figure S1B) indicating that in general, Tregs of both strains were equipped with receptors allowing them to enter the infected tissue.

### In the Draining Lymph Node Treg Cell Numbers were Reduced in B6.CCR6^−/−^ Mice While Th17 Cell Numbers Remained Unchanged after *L. major* Infection

**Figure 2:**
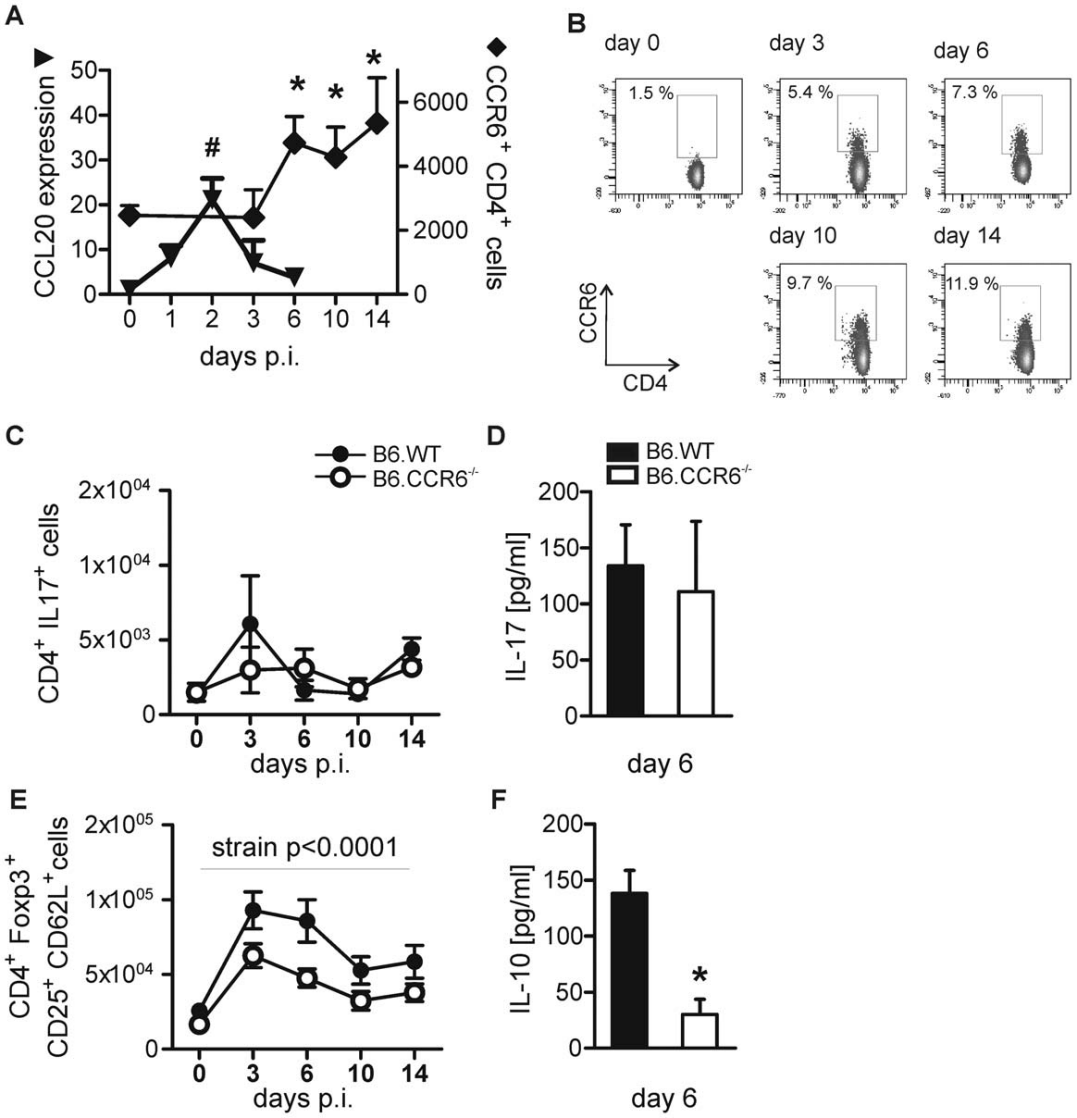
Th17 cell numbers remained unchanged and Treg cells increased in B6.CCR6^−/−^ mice. Mice were infected s.c. with 3×10^6^ *L. major* promastigotes. (A–B) The number of CD4^+^CCR6^+^ cells and CCL20 mRNA expression in the draining lymph nodes of B6.WT mice were determined. (A) CCL20 expression in the draining lymph node was quantified by RT-PCR. The relative expression of CCL20 on d 1 to d 6 compared to d 0 is shown (n = 3 mice each time point, data represent one experiment). The number of CD4^+^CCR6^+^ cells was determined by flow cytometry. Live cells were gated on CD4^+^CCR6^+^ cells. The number of CD4^+^CCR6^+^ cells is presented (d 0–10, n = 9 mice; day 14, n = 6 mice per time point, pooled data of three independent experiments, */# p<0.05 compared to d 0). (B) A representative dot plot analysis shows the percentage of CCR6^+^ cells within the CD4 cell gate. (C–F) Lymph node cells were harvested at the indicated time points. (C) Cells were stimulated *in vitro* with PMA and ionomycin in the presence of BFA and analyzed by flow cytometry. Events were gated on CD4^+^ IL-17^+^ events and the absolute number of cells was calculated. (E) Cells were isolated from draining lymph nodes and immediately stained for CD4, CD25, CD62L, and Foxp3. Live cells were gated on CD4^+^ Foxp3^+^ cells and further subdivided based on the expression of CD25 and CD62L. The number of CD4^+^ Foxp3^+^ CD25^+^ CD62L^+^ is given (d 0–10, n = 6 mice; day 14, n = 3 mice per time point and genotype, pooled data from two independent experiments). (D and F) To quantify cytokine production lymph node cells from B6.WT or B6.CCR6^−/−^ mice were cultured for 4 d in the presence of LAg and supernatants were collected. The concentrations of IL-17 (D) and IL-10 (F) are shown (n = 6 mice per genotype, pooled data of two independent experiments, *p<0.05).

Six days after infection inflammatory T cells were more frequent in the skin of B6.CCR6^−/−^ than in B6.WT mice indicating a marked activation of those cells in the early phase of infection. The lymph node is the major site of T cell priming. To reveal the role of CCR6 expressing cells in the anti-*L. major* immune response we monitored the expression of the CCR6 ligand CCL20 and the number of CD4^+^CCR6^+^ cells in the draining lymph nodes during the early phase of infection in B6.WT mice. CCL20 expression increased on d 2 of infection (Figure 2A). Consequently, CD4^+^CCR6^+^ cell numbers started to rise from d 6 of infection in the lymph node (Figure 2A and B).

Having detected an increase in CCR6^+^ cells in the draining lymph nodes of B6.WT mice we further characterized those CCR6-expressing cells. Although CCR6 expression is a prerequisite of Th17 cells, the level of Th17 cells was not different between B6.WT and B6.CCR6^−/−^ mice (Figure 2C). In both strains the highest frequency of Th17 cells was measured in non-infected mice and declined after infection (Figure S2D). Their absolute numbers remained unchanged during the first 2 weeks of infection, while other Th cell populations increased. Consequently, lymph node cells of infected B6.WT and B6.CCR6^−/−^ mice produced equal amounts of IL-17 after antigen-specific re-stimulation *in vitro* (Figure 2D).

Next, we monitored Treg cells in the draining lymph nodes. After infection, Treg cell numbers increased (Figure 2E). The number of total Foxp3^+^ Treg cells (data not shown) as well as Foxp3^+^ CD25^+^CD62L^+^ Treg cells in the draining lymph node was lower in B6.CCR6^−/−^ compared to B6.WT mice (Figure 2E). This finding corresponded with the lower amount of IL-10 produced by lymph node cells after re-stimulation with LAg *in vitro* (Figure 2F). Of note, the amount of TGFβ measured in antigen-stimulated lymph node cell cultures was not different between B6.WT and B6.CCR6^−/−^ mice (data not shown).

### B6.CCR6^−/−^ Mice Developed a Protective Th1 Immune Response but also Showed Enhanced Th2 Cytokine Expression

**Figure 3:**
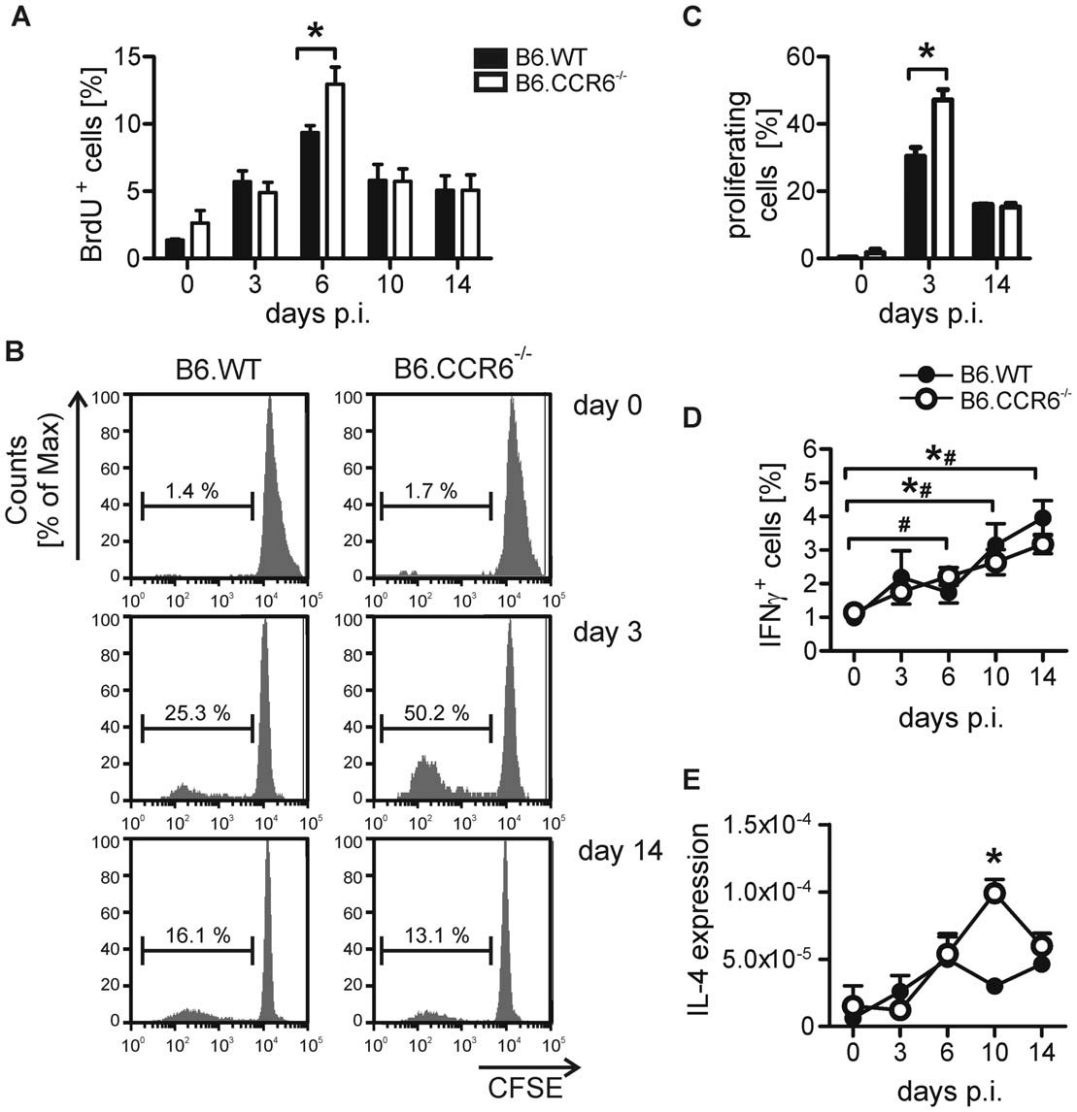
Th cell proliferation was increased in B6.CCR6^−/−^ mice. B6.WT and B6.CCR6^−/−^ mice were infected s.c. with 3×10^6^ *L. major* promastigotes. Lymph node cells were isolated at the indicated time points and cells were analyzed by flow cytometry. (A) Mice were given BrdU in drinking water three days before analysis. Cells stained for CD4 and BrdU were gated on CD4^+^ cells. The percentage of BrdU^+^ events is shown (n = 3 per time point and genotype, data represent one experiment). (B–C) Lymph node cells were labeled with CFSE and stimulated with LAg *in vitro* for 4 d. Cells were gated on CD4^+^ events. (B) One representative histogram plot of proliferating cells is shown. (C) The percentage of proliferating cells is shown (n = 3 mice per time point and genotype, data represent one experiment, *p<0.05). (D) Lymph node cells were stimulated with PMA and ionomycin in the presence of BFA. Live cells were gated on CD4^+^ events. The percentage of IFNγ^+^ cells is shown (d 0–10, n = 9 mice; day 14, n = 6 mice per time point and genotype, pooled data of three independent experiments, *p<0.05, B6.WT; ^#^ p<0.05, B6.CCR6^−/−^). (E) For analysis of IL-4 expression lymph nodes were harvested, total RNA was isolated and IL-4 mRNA was quantified by RT-PCR. Data show the expression of IL-4 mRNA relative to β-actin mRNA (n = 3 mice per time point and genotype, data represent one experiment, *p<0.05).

Resolution of *L. major* infection in C57BL/6 mice is critically dependent on the developing of a Th1 immune response. To test whether the early reduction in Treg cells affected the regulation of Th1 immune responses, we monitored Th cell proliferation and cytokine production between d 3 and d 14 in the lymph node. The initial event in T cell activation is the proliferation of T cells starting around d 3 after antigen contact [22]. This is followed by a differentiation phase indicated by the development of cytokine-producing effector cells. In this study proliferation was reflected by a continuous increase in lymph node cell numbers after infection (Figure S2A). The increase in cell numbers was comparable between B6.WT and B6.CCR6^−/−^ mice. Monitoring CD4 T cell proliferation *in vivo* by BrdU incorporation, we observed an early expansion of Th cells between d 3 and d 6 and a reduction in the proliferation afterwards (Figure 3A). Proliferation of CD4 T cells followed the same kinetics in both strains. However, a higher proliferation rate was seen in B6.CCR6^−/−^ mice on d 6. The finding was confirmed by analysis of *in vitro* proliferation after antigen-specific re-stimulation of lymph node cells. Cells of B6.CCR6^−/−^ mice re-stimulated on d 3 after *L. major* inoculation proliferated stronger than those of B6.WT mice (Figure 3B and C), although the cells divided with similar kinetics in both strains. These data indicate that CD4 cells were activated more rapidly in B6.CCR6^−/−^ mice. In both strains the frequency of IFNγ-producing CD4 cells increased steadily (Figure 3D). However, in B6.WT mice the frequency of IFNγ-producing cells did not increase significantly until d 10 of *L. major* infection; whereas a slight but significant increase in the frequency of IFNγ-producing CD4 cells was already detected on d 6 post infection (p.i.) in B6.CCR6^−/−^ mice (Figure 3D) while the proportion of total CD4 cells in the lymph nodes was not different in the two strains (Figure S2B). Next, we tested whether the Th2 immune response was also affected by the absence of CCR6. Initially, an induction of IL-4 expression was detected in both strains. While B6.WT started to down-modulate IL-4 expression on d 10 the decrease in IL-4 expression was delayed in B6.CCR6^−/−^ mice (Figure 3E).

### Lack in CCR6 Led to Increased CD8 T Cell Proliferation and Cytokine Production

**Figure 4:**
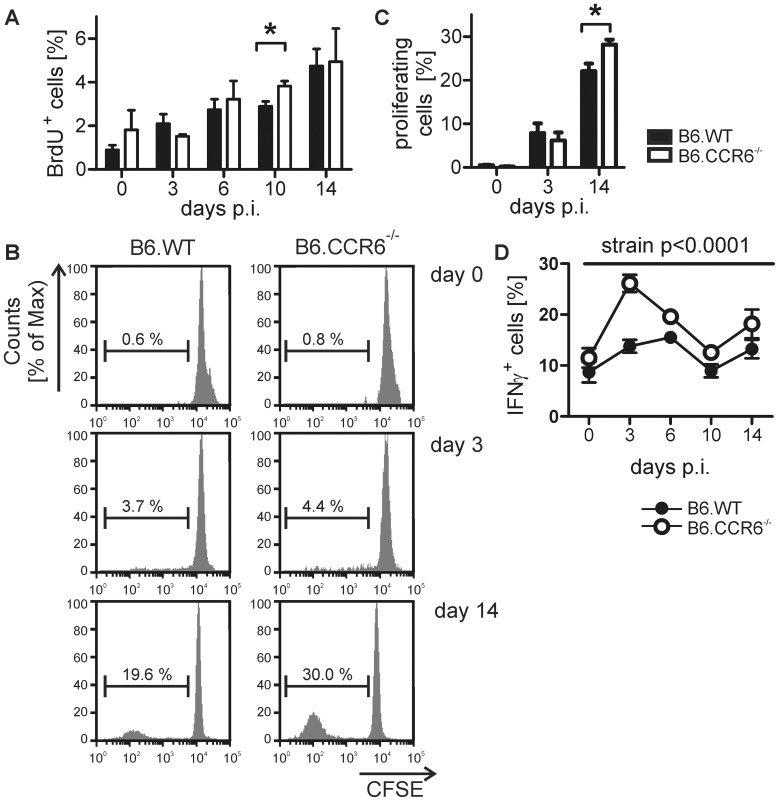
Proliferation and cytokine production by CD8 cells during the early phase of *L. major* infection. B6.WT and B6.CCR6^−/−^ mice were infected s.c. with 3×10^6^ *L. major* promastigotes. Lymph node cells were isolated at the indicated time points and cells were analyzed by flow cytometry. (A) Mice were given BrdU in drinking water three days before analysis. Lymph node cells stained for CD8 and BrdU were gated on CD8^+^ cells. The percentage of BrdU^+^ events is shown (n = 3 per time point and genotype, data represent one experiment, *p<0.05). (B) Lymph node cells were labeled with CFSE and stimulated with LAg *in vitro* for 4 days. Cells were gated on CD8^+^ events. One representative histogram plot of proliferating cells is shown. (C) The percentage of proliferating cells is shown (n = 3 mice per time point and genotype, data represents one experiment). (D) Lymph node cells were stimulated with PMA and ionomycin in the presence of BFA. Live cells were gated on CD8^+^ events. The percentage of IFNγ^+^ cells is shown (d 0–10, n = 6 mice; day 14, n = 3 mice per time point and genotype, pooled data of two independent experiments).

As shown after low dose infection with *L. major,* enhanced IL-4 expression is followed by a compensatory IFNγ response by CD8 cells [23]. Thus, we were interested whether the observed increase in IL-4 expression in B6.CCR6^−/−^ mice corresponded with enhanced CD8 cell proliferation and IFNγ production. In contrast to CD4 cells lymph node-residing CD8 cells proliferated steadily until d 14, the latest time point tested. Enhanced CD8 cell proliferation was observed *in vivo* on d 10 in CCR6^−/−^ mice compared to B6.WT mice (Figure 4A). Monitoring antigen-induced proliferation *in vitro* on d 14 also revealed an increased proliferative capacity of CD8 cells derived from CCR6-deficient mice, indicating a more activated state of CD8 cells in mice lacking CCR6 expression (Figure 4B and C). Additionally, we detected a higher frequency of IFNγ-producing CD8 cells in B6.CCR6^−/−^ mice during the observation period of 14 days (Figure 4D) while the percentage of total CD8 cells was comparable in both strains (Figure S2C). Activation of CD8 cells normally is delayed compared to CD4 cell activation [24]. However, mice lacking CCR6 showed an increased frequency of IFNγ-producing CD8 cells already on d 3 of infection, indicating a lower activation threshold or the absence of regulatory mechanisms in B6.CCR6^−/−^ mice.

### Increased Inflammatory Response in CCR6-deficient Mice After 4 Weeks of Infection is Indicated by Enhanced Th1 Cytokine Production in the Lymph Node

**Figure 5:**
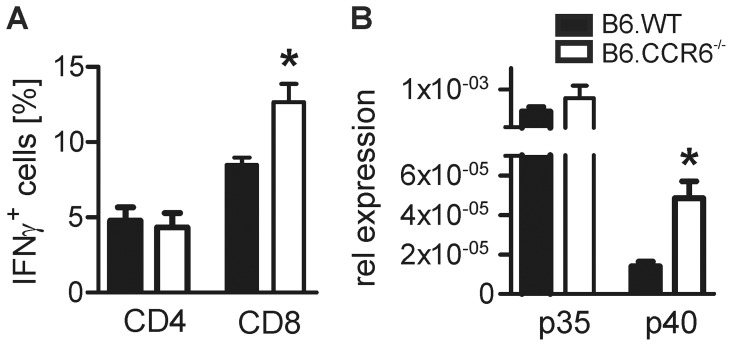
IFNγ-producing CD4^+^ and CD8^+^ cells and IL-12 expression at the peak of infection. Lymph nodes of B6.WT or B6.CCR6^−/−^ mice were harvested after 4 weeks of infection with *L. major* parasites and cells were isolated. (A) Lymph node cells restimulated *in vitro* with PMA and ionomycin in the presence of BFA were stained for CD4, CD8 and IFNγ and analyzed by flow cytometry. Cells were gated on CD4^+^ or CD8^+^ events. The percentage of IFNγ^+^ events of either CD4^+^ or CD8^+^ events is shown (n = 3 mice, data represent one experiment, *p<0.05). (B) RNA was isolated from draining lymph node tissue and IL-12p35 and IL-12p40 mRNA expression was quantified by RT PCR. Data show expression of p35 and p40 mRNA relative to β-actin mRNA (n = 3 mice, data represent one experiment, *p<0.05).

In B6.CCR6^−/−^ mice the early phase of *L. major* infection was characterized by a decrease in Treg cells and an increased frequency of IFNγ-producing CD8 cells in the lymph nodes. To investigate long-term consequences of the early hyper-inflammatory response detected in B6.CCR6^−/−^ mice, we investigated the T cell activation state at the peak phase of infection after 4 weeks. The percentage of IFNγ-producing CD4 cells was comparable in both strains showing that mice effectively mounted a Th1 immune response (Figure 5A). The frequency of IFNγ-producing CD8 cells was higher in B6.CCR6^−/−^ compared to B6.WT mice as seen already in the early phase of infection (Figure 5A). Consistently, higher levels of IL12p40 mRNA in B6.CCR6^−/−^ mice compared to B6.WT mice 4 weeks after infection indicated an over-activation of the Th1/Tc1 immune pathway (Figure 5B). IL12p35 mRNA was not increased in infected versus non-infected mice (data not shown) and also no difference was detected in IL12p35 mRNA between the two strains (Figure 5B). As the p40 subunit is shared by IL-12 and the Th17 cell-supporting cytokine IL-23, we also monitored mRNA expression of IL23p19. Expression of p19 mRNA was induced at the early phase of infection on d 3 and d 6. However, after 4 weeks of infection p19 mRNA was below the detection limit (data not shown) arguing against a critical role of Th17 cells during *L. major* infection in resistant C57BL/6 mice.

### In CCR6-deficient Mice Increased Local Inflammation Paralleled with Abundant Numbers of Gr1^+^ Cells in the Lesions After 5 Weeks of Infection

**Figure 6:**
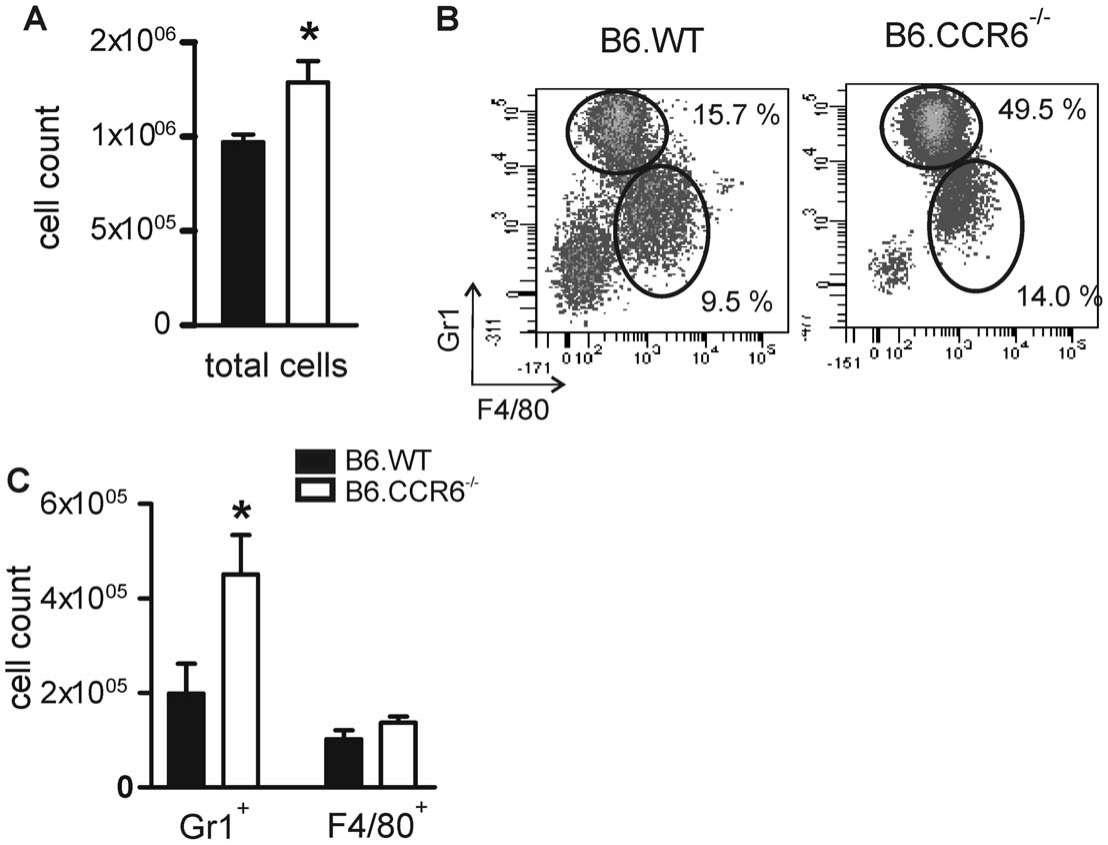
Increase in granulocytes at the site of infection in B6.CCR6^−/−^ mice. Mice were infected with 3×10^6^ *L. major* promastigotes. Cells were isolated from footpad lesions of B6.WT or B6.CCR6^−/−^ mice on day 35 after infection. (A) The total cell count was determined by trypan blue exclusion. (B–C) Cells were stained for CD11b, Gr1 and F4/80 and analyzed by flow cytometry. Cells were gated on CD11b^+^ cells. (B) The percentage of Gr1^+^ and F4/80^+^ cells is shown in a representative dot plot. (C) The total number of Gr1^+^ and F4/80^+^ cells are shown (n = 4 mice per genotype, data represents one experiment, *p<0.05).

At the peak of infection B6.CCR6^−/−^ mice developed more severe lesions than B6.WT mice. B6.CCR6^−/−^ mice showed a similar parasite load as B6.WT mice in the footpad lesions. Hence, we were questioning whether the increased inflammatory response observed in the lymph nodes might result in a more pronounced inflammation at the site of infection in CCR6-deficent mice. Analysis of the cellular composition at the lesion site after 5 weeks of infection revealed increased numbers of total leukocytes in the footpads of CCR6^−/−^ mice (Figure 6A). A detailed analysis of the infiltrating cells showed that those cells consisted mainly of macrophages and granulocytes (Figure 6B). While macrophage counts were comparable between B6.WT and B6.CCR6^−/−^ mice, a significant increase in Gr1^+^ granulocytes was detected in mice lacking CCR6 (Figure 6C).

## Discussion

It is still under debate whether Th17 cells and Treg cells contribute to protective immunity during the primary *L. major* infection in resistant mice. As the chemokine receptor CCR6 is shared by Th17 and Treg cells we considered B6.CCR6^−/−^ mice as an ideal model to investigate the role of Treg and Th17 cells during *L. major* infection. In this study we report that increased local tissue inflammation in B6.CCR6^−/−^ mice correlated with reduced numbers of Treg cells during *L. major* infection. The reduction in Treg cells seemed to play a major role in the increased inflammatory immune response observed in mice lacking CCR6.

CCR6-expressing Th cells have been intensively studied in autoimmune diseases and chronic inflammations. Several *in vitro* studies have analyzed the paradox of CCR6 being shared by pro-inflammatory Th17 cells and immune-suppressive Treg cells [7], [8], [9]. It turned out that mainly the cytokine environment, strength of activation signals, and the presence of microbial stimuli determine whether CCR6^+^ Th cells differentiate in one or the other direction [10], [11], [25]. However, the spatial requirements are often underestimated by *in vitro* studies and animal studies may help to clarify this issue. Chemokines guide leukocytes through lymphatic and non-lymphatic tissues. Thus, it is likely that the presence of specific chemokine receptors on Th subsets influences their function [13]. Our study revealed that CCR6 expression is required for Treg cell recruitment in the draining lymph node early during *L. major* infection to prevent excessive immune cell activation and tissue damage, whereas Th17 cell migration was not affected by the absence of CCR6 in our model. Th17 cells link innate and adaptive immunity and are present as a thymus-derived population of natural Th17 cells [26]. While they are equipped to react quickly to invading pathogens and are thus pivotal for the clearance of bacterial infections [27], their function during *L. major* infection is still controversial. Production of Th17-related cytokines correlated with protection against visceral leishmaniasis caused by infection with *L. donovani* in humans [28]. However, after *L. major* infection Th17 cell numbers increase only transiently in resistant B6.WT mice, whereas Th1 cells are the predominant cell type in the long-lasting, anti-parasitic immune response [29]. Thus, Th17 cells seem to play a redundant role during *L. major* infection.

While Th1 cell-mediated immunity is crucial for the resolution of *L. major* infection IL-4-producing cells also emerge in resistant C57BL/6 mice [30], [31]. Treg cells were shown to be responsible for the suppression of the early Th2 and the stabilization of the protective Th1 response [32]. As transient IL-4 expression has been demonstrated to trigger compensatory CD8-mediated IFNγ responses [23], it is likely that the inability of B6.CCR6^−/−^ mice to recruit sufficient numbers of Treg cells prolonged Th2 cytokine expression after *L. major* infection and supported further inflammation in B6.CCR6^−/−^ mice. Even though CD8 cells seem to be dispensable for overall protection against the infection [33], [34], [35], early IFNγ production by CD8 cells was proposed to favor the Th1 immune response during leishmaniasis [36]. The fact that CCR6-deficient mice were able to mount a protective Th1 response provides a likely mechanism explaining the healing phenotype of those mice. In line with that, CCR6^−/−^ mice showed a tendency towards reduced parasite load in the draining lymph nodes on day 10 and 28. However, exuberant CD8 T cell activation has been demonstrated to enhance immune pathology during *L. major* infection [24]. In humans, destructive forms of cutaneous leishmanasis are associated with higher frequencies of CD8 cells and high levels of IFNγ in lymph node tissue as well as at the site of infection [37], [38], [39]. In B6.CCR6^−/−^ mice increased capability of CD8 cells to produce IFNγ also correlated with higher levels of IL12p40 expression at the peak of infection. It seems likely that high numbers of CD8 cells caused a sustained IL-12 response in dendritic cells as it has been demonstrated after ovalbumin vaccination [40].

An important role for the limitation of immune pathology has been attributed to Treg cells in various parasitic infections [32], [41], [42]. During *L. major* infection the early decrease in Treg cells paralleled with an increased Th cell proliferation in the lymph nodes and a subsequent increased frequency of inflammatory T cells in the skin in CCR6^−/−^ mice. While CCR6 has been demonstrated to recruit Treg cells to the inflamed tissues in several autoimmune diseases [43], [44], in leishmaniasis the receptor seems to be important for Treg cell accumulation in the lymph nodes where they regulate the inflammatory response. This assumption is supported by a recent study in a model of contact hypersensitivity in CCR4-deficient mice [45]. CCR4 is also expressed on inflammatory and on regulatory Th cell subtypes. Despite showing comparable numbers of Treg cells in the inflamed skin the study provides evidence that impaired Treg cell function resulted in a more severe local inflammation in CCR4^−/−^ mice.

The lymph node, as the major site of lymphocyte activation, provides information about immune cell responses present in the infected host. However, the effectiveness of anti-parasitic effector mechanisms is reflected at the site of infection. We have previously shown that macrophages derived from B6.CCR6^−/−^ mice were able to up-regulate iNOS expression, allowing them to eradicate *L. major* parasites [18]. We now add data demonstrating that recruitment of effector T cells and macrophages into the skin was not affected by the absence of CCR6. Consequently, the clearance of *L. major* parasites in the lymph nodes and at the site of infection was comparable between B6.CCR6^−/−^ and B.6.WT mice. Interestingly, granulocyte recruitment worked even better in mice lacking CCR6. Despite the fact that CCR6 is crucial for Th17 cell migration into the skin, Th17 cells were more frequent in the lesions at early time points. Other chemokine receptors, like CCR4, are likely candidates contributing to the cutaneous migration of Th17 cells in those mice [46]. Nevertheless, the early accumulation of Th17 cells at the site of infection might condition the lesion for the subsequent attraction of granulocytes as it has been shown recently in susceptible BALB/c mice [17], [47], [48]. At the peak of infection Th17 cells were rather rare in the skin. At that time point other molecules, e.g. TNF and IL-1, might compensate the lack of IL-17-supporting long-term accumulation of granulocytes [49], [50]. As Th17 cell-mediated granulocyte recruitment and activation in the chronic phase of infection correlates with tissue damage [51], the recruitment of granulocytes might favor further tissue damage and promote local inflammation following *L. major* infection in B6.CCR6^−/−^ mice.

In this study we provide evidence that Treg cells can be attributed as a major regulatory subtype preventing excessive production of IFNγ and reducing local inflammation after infection in CCR6^−/−^ mice. Our data argue for CCR6 playing a role in Treg cell rather than Th17 cell recruitment during parasitic infections that depend on cell-mediated immune response as the predominant protective immune mechanism.

## Supporting Information

Figure S1 Cytokine producing T cells in the footpad lesions. Mice were infected s.c. with 3×10^6^ *L. major* promastigotes. Footpads were dissected at the indicated time points and cells were isolated. (A) Cells were stained for CD4, CD8, IFNγ and IL-17. Live cells were gated for CD4^+^ (red) and CD8^+^ (green) cells. The mean percentage (± SEM) of IFNγ^+^ and IL-17^+^ cells is shown. (B) Cells were harvested from the footpads on day 28 of infection and stained for CD4, Foxp3, CD25, and CD103. Cells were gated on CD4^+^ Foxp3^+^ cells. The mean percentage (± SEM) of either CD25^+^ or CD103^+^ cells of CD4^+^ Foxp3^+^ cells is shown in the histogram plot (n = 4 per time point and genotype, one experiment).
(TIF)

Figure S2 T cell composition in lymph nodes of B6.WT and B6.CCR6^−/−^ mice during *L. major* infection. Mice were infected as described and lymph node cells were isolated. (A) Live cells were counted by trypan blue exclusion. The number of total lymph node cells is shown (n = 12–18 mice per time point and genotype, pooled data of 6 independent experiments). (B-C) Lymph node cells stained for CD4 and CD8 were analyzed by flow cytometry. The percentage of either CD4 (B) or CD8 (C) cells in the lymph nodes is shown (n = 6–9 mice for each time point and genotype, pooled data of 3 independent experiments). (D–E) Cells were gated on CD4^+^ (D) or CD8^+^ (E) cells and the percentages of IFNγ^+^ and IL-17^+^ cells were determined. A representative dot plot analysis is shown (n = 3–6 mice per time point and genotype; two independent experiments).
(TIF)
