# Supplementary figures and images for: An Early Reduction in Treg Cells Correlates with Enhanced Local Inflammation in Cutaneous Leishmaniasis in CCR6-Deficient Mice
Source: PLoS One. 2012 Sep 28;7(9):e44499. doi: 10.1371/journal.pone.0044499 (PMC3460949; doi:10.1371/journal.pone.0044499)

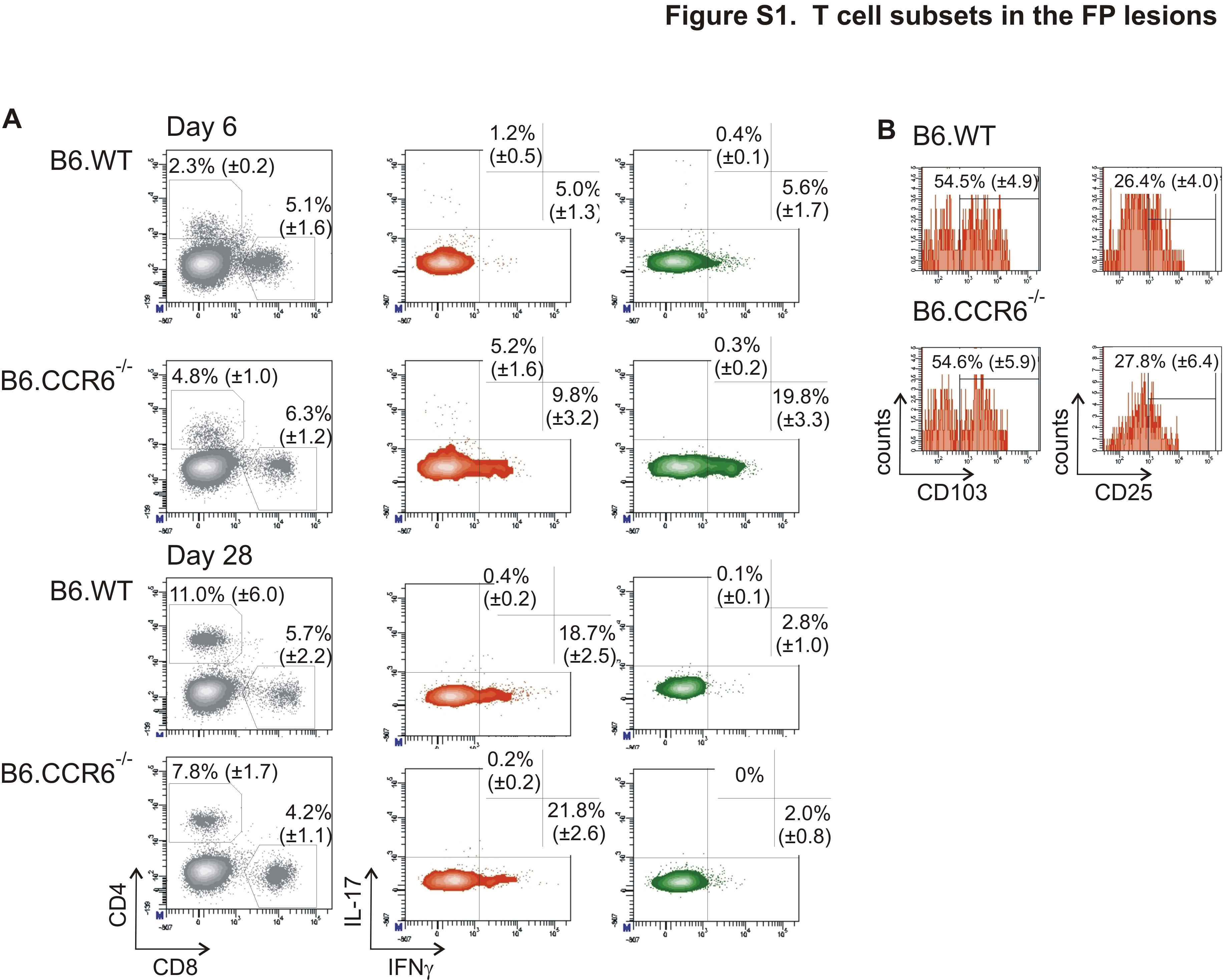

Supplement: Figure S1 — Cytokine producing T cells in the footpad lesions. Mice were infected s.c. with 3×10^6^ L. major promastigotes. Footpads were dissected at the indicated time points and cells were isolated. (A) Cells were stained for CD4, CD8, IFNγ and IL-17. Live cells were gated for CD4+ (red) and CD8+ (green) cells. The mean percentage (± SEM) of IFNγ+ and IL-17+ cells is shown. (B) Cells were harvested from the footpads on day 28 of infection and stained for CD4, Foxp3, CD25, and CD103. Cells were gated on CD4+ Foxp3+ cells. The mean percentage (± SEM) of either CD25+ or CD103+ cells of CD4+ Foxp3+ cells is shown in the histogram plot (n = 4 per time point and genotype, one experiment). (TIF) [file pone.0044499.s001.tif]

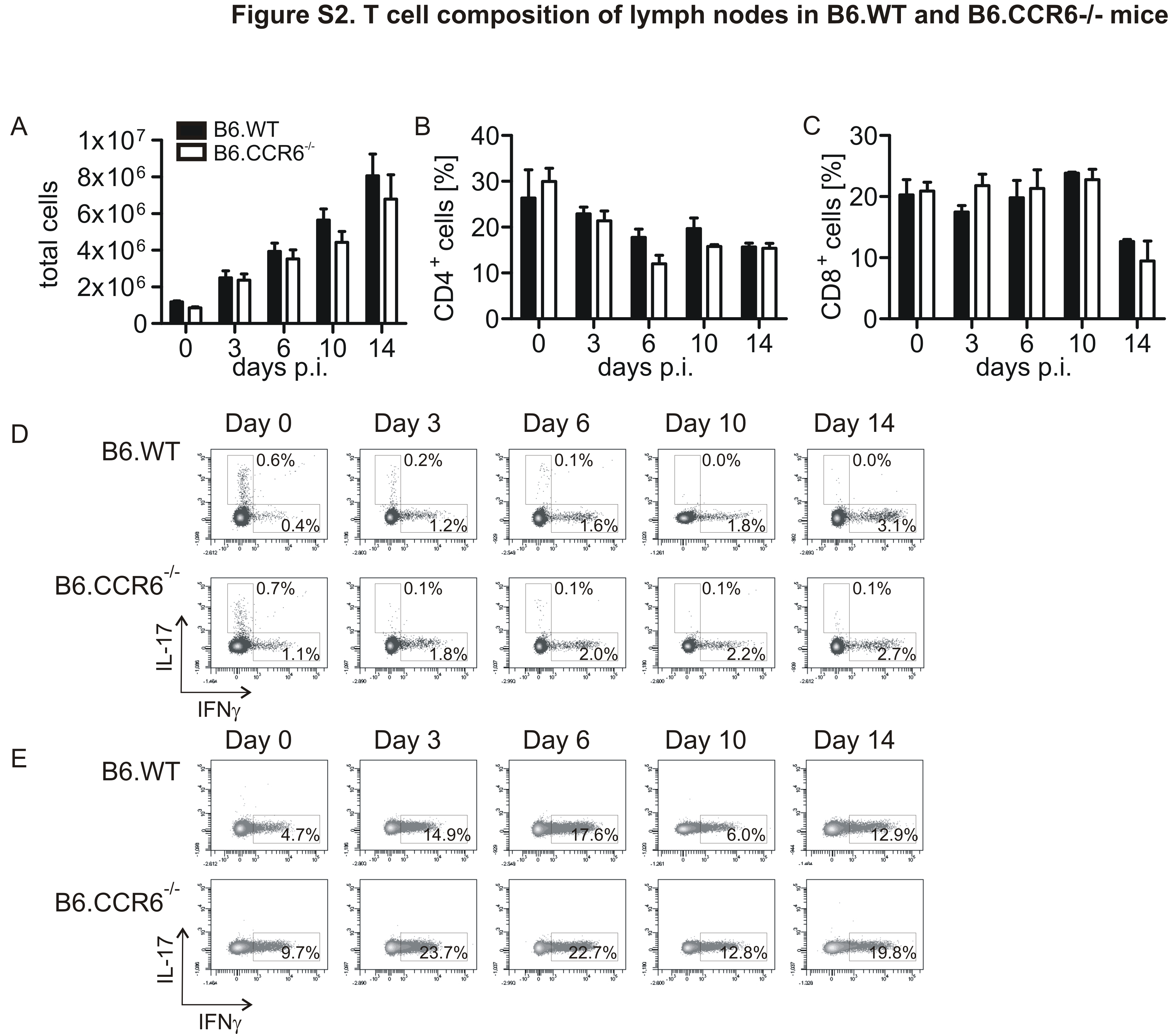

Supplement: Figure S2 — T cell composition in lymph nodes of B6.WT and B6.CCR6−/− mice during L. major infection. Mice were infected as described and lymph node cells were isolated. (A) Live cells were counted by trypan blue exclusion. The number of total lymph node cells is shown (n = 12–18 mice per time point and genotype, pooled data of 6 independent experiments). (B-C) Lymph node cells stained for CD4 and CD8 were analyzed by flow cytometry. The percentage of either CD4 (B) or CD8 (C) cells in the lymph nodes is shown (n = 6–9 mice for each time point and genotype, pooled data of 3 independent experiments). (D–E) Cells were gated on CD4+ (D) or CD8+ (E) cells and the percentages of IFNγ+ and IL-17+ cells were determined. A representative dot plot analysis is shown (n = 3–6 mice per time point and genotype; two independent experiments). (TIF) [file pone.0044499.s002.tif]
